# Neurotoxic Effect of Ethanolic Extract of Propolis in the Presence of Copper Ions is Mediated through Enhanced Production of ROS and Stimulation of Caspase-3/7 Activity

**DOI:** 10.3390/toxins11050273

**Published:** 2019-05-15

**Authors:** Vedrana Radovanović, Josipa Vlainić, Nikolina Hanžić, Petra Ukić, Nada Oršolić, Goran Baranović, Maja Jazvinšćak Jembrek

**Affiliations:** 1 Division of Molecular Medicine, Rudjer Boskovic Institute, 10000 Zagreb, Croatia; vedranaradovanovic@yahoo.com (V.R.); josipa.vlainic@irb.hr (J.V.); Nikolina.Hanzic@irb.hr (N.H.); 2 Department of Animal Physiology, Faculty of Science, University of Zagreb, 10000 Zagreb, Croatia; petrauki@gmail.com (P.U.); norsolic@yahoo.com (N.O.); 3 Division of Organic Chemistry and Biochemistry, Rudjer Boskovic Institute, 10000 Zagreb, Croatia; Goran.Baranovic@irb.hr; 4 Department of Psychology, Catholic University of Croatia, Ilica 242, 10000 Zagreb, Croatia

**Keywords:** ethanolic extract of propolis, copper toxicity, p53, p38 and JNK signaling, P19 neurons

## Abstract

Elevated amounts of copper are considered to be contributing factor in the progression of neurodegenerative diseases as they promote oxidative stress conditions. The aim of our study was to examine the effects of ethanolic extract of propolis (EEP) against copper-induced neuronal damage. In cultured P19 neuronal cells, EEP exacerbated copper-provoked neuronal cell death by increasing the generation of reactive oxygen species (ROS) and through the activation of caspase-3/7 activity. EEP augmented copper-induced up-regulation of p53 and Bax mRNA expressions. Neurotoxic effects of EEP were accompanied by a strong induction of glyceraldehyde 3-phosphate dehydrogenase (GAPDH) expression and decrease in the expression of c-fos mRNA. SB203580, an inhibitor of p38 mitogen-activated protein kinase (MAPK) prevented detrimental effects of EEP, whereas SP600125, an inhibitor of c-Jun N-terminal kinase (JNK), exacerbated EEP-induced neuronal cell death. Quercetin, a polyphenolic nutraceutical, which is usually present in propolis, was also able to exacerbate copper-induced neuronal death. Our data indicates a pro-oxidative and apoptotic mode of EEP action in the presence of excess copper, wherein ROS/p53/p38 interactions play an important role in death cascades. Our study also pointed out that detailed pharmacological and toxicological studies must be carried out for propolis and other dietary supplements in order to fully recognize the potential adverse effects in specific conditions.

## 1. Introduction

Oxidative stress is a condition that is characterized by increased accumulation of reactive oxygen species (ROS) that overwhelm the intracellular antioxidative defense. Although ROS have an important physiological function, mainly as signaling molecules in redox-sensitive signaling pathways, their accumulation has harmful effects on neuronal viability. Previous studies have demonstrated that oxidative stress underlies pathological processes in neurodegeneration, contributing to the development and progression of various disorders such as Alzheimer’s and Parkinson’s disease [1,2]. 

Oxidative stress can be induced by increased accumulation of copper ions. Copper is essential for electron-transfer reactions in diverse biological processes, but in certain conditions (environmental pollution, accidents, excess dietary intake, inborn errors of copper metabolism and specific medical cases), it may accumulate in unbound forms and trigger ROS-generating reactions [3]. This increase in ROS may further induce oxidative damage to biological macromolecules and modulate intracellular transduction pathways, compromising neuronal function and viability [4,5]. Besides, copper itself may bind to thiol (-SH) groups of various proteins and disturb their functions, which additionally threatens cellular homeostasis [6]. Ultimately, ROS-initiated deleterious events end in neuronal death. In a cell type-, concentration- and time-dependent manner, copper-induced signaling pathways may trigger intrinsic and extrinsic caspase-dependent and caspase-independent apoptosis [5,7,8]. Elevated concentrations of copper are reported in patients suffering from various neurodegenerative disorders and copper dyshomeostasis is considered as a contributing factor to the onset and progression of pathological processes implicated in neurodegeneration [3,9,10]. Accordingly, therapeutic strategies based on the prevention of copper-mediated oxidative stress conditions could be a promising approach in neuroprotection.

Dietary supplements of natural origin that possess antioxidative activity are often taken as an adjuvant therapy intended to prevent or delay oxidative stress-induced injury in neurodegeneration. It is believed that they may improve well-being by re-establishing redox homeostasis and exert beneficial effects on cognitive and motor impairments [11]. There is, however, compelling evidence to suggest that natural agents with anti-oxidative activity may also exert pro-oxidative effects, particularly in the presence of transition metal ions that initiate a Fenton reaction and ROS generation [11,12,13]. Propolis, a heterogeneous resinous product collected by honey-bees, is a popular natural remedy that can be easily obtained at many health food and drug stores. Due to its well-documented biological effects, such as being antioxidant, anti-inflammatory, immunomodulatory, antimicrobial and anticancer, propolis and its extracts are used to treat various clinical conditions in traditional and modern medicine [14,15]. Previous studies have demonstrated neuroprotective actions of methanol, ethanol or water extracts of propolis against oxidative stress-induced damage in different cellular models and brain tissue. Its beneficial effects were mostly attributed to free-radical scavenging activity of propolis components. In particular, methanol extracts of propolis protected non-differentiated SH-SY5Y neuroblastoma cells against hydrogen peroxide (H_2_O_2_)-induced oxidative stress by suppressing ROS generation [16], whereas ethanol and water extracts of propolis were effective against H_2_O_2_-induced death of PC12 cells differentiated into mature neurons. Ethanolic extracts were also able to reduce brain infarction at 24 h after the occlusion in a model of focal cerebral ischemia [17].

To our knowledge, effects of propolis have never been studied in copper-induced oxidative neuronal injury. Hence, the aim of our study was to investigate the effects of ethanolic extract of propolis (EEP) on copper-provoked neurodegeneration and to clarify the molecular and cellular mechanisms of its action.

## 2. Results

**Figure 1:**
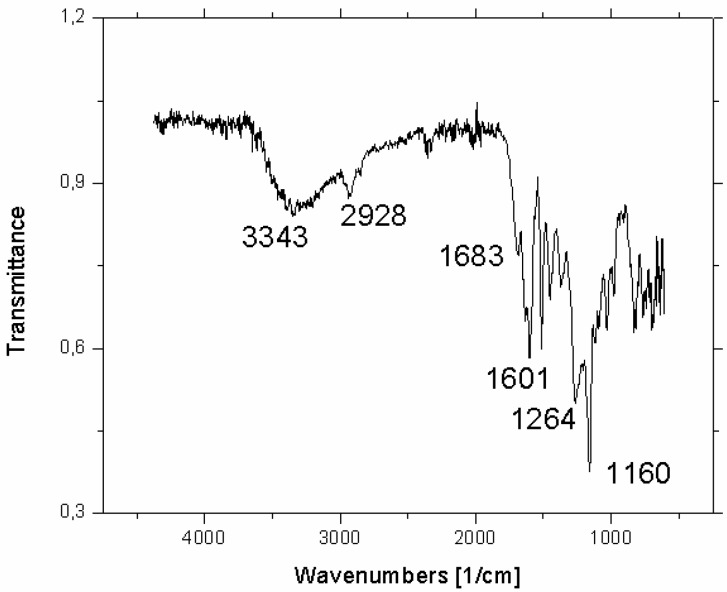
Attenuated total reflectance (ATR) FTIR spectrum of propolis resin with the wavenumbers of the dominant bands depicted.

Authenticity of the propolis resin sample was confirmed by the FTIR spectra and the results are depicted in Figure 1. 

The spectrum confirms the presence of various propolis compounds but due to their enormous number (see Tables 2 and 3 in ref. [18]), it is impossible to identify any of these compounds individually. Nonetheless, some qualitative information can be extracted; and according to Franca et al. [19], the strong absorption at 1683 cm^−1^ could be due to C=O stretchings of flavonoids and lipids. The bands at 1634, 1602 and 1513 cm^−1^ can be attributed to aromatic rings, while the strongest absorption at 1160 cm^−1^ could be assigned to hydroxyl group vibrations, the C-O stretchings and C-OH bendings.

### 2.1. Propolis Exacerbated Copper-Induced Neuronal Injury in P19 Neuronal Cells

**Figure 2:**
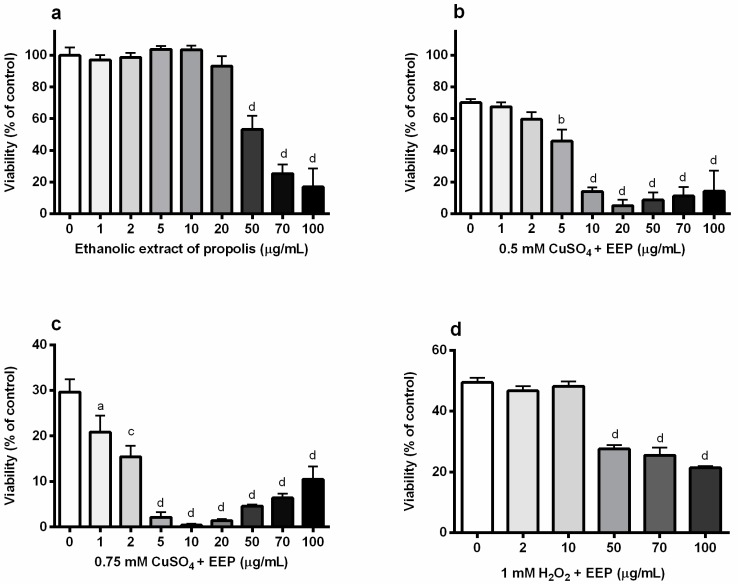
Ethanolic extract of propolis (EEP) exacerbated copper-induced neuronal death in P19 neurons. P19 neurons were obtained from the mouse embryonal carcinoma cells in the presence of all-trans retinoic acid (ATRA). Measurements are performed at 24 h from the beginning of treatment with copper and EEP. (**a**) P19 neurons were incubated with increasing concentrations of EEP (1–100 µg/mL) for 24 h and their viability was assessed by an MTT assay. Both non-toxic and toxic concentrations of EEP augmented neuronal death induced by exposure to 0.5 mM CuSO_4_ (**b**) and 0.75 mM CuSO_4_ (**c**). This effect of EEP on the reduction of the neuronal viability was not evident when oxidative stress was induced by exposure to 1 mM H_2_O_2_ (**d**). Data are expressed as means ± SEM from four to six independent experiments performed in quadruplets. ^a^ *P* < 0.05; ^b^ *P* < 0.01; ^c^ *P* < 0.001 and ^d^ *P* < 0.0001 one-way ANOVA followed by Dunnett’s multiple comparison test.

At first, we studied the effect of EEP on the viability of differentiated P19 neuronal cells. As expected, EEP reduced survival only when present at very high concentrations, from 50 µg/mL and above (Figure 2A). We previously demonstrated that copper in a dose-dependent manner decreases the viability of P19 neuronal cells [5]. In experiments aimed at resolving the effect of EEP on the viability of P19 cells exposed to excess copper (0.5 mM and 0.75 mM CuSO_4_), we found that small, non-toxic concentrations of EEP could exacerbate the neurotoxic effects of copper. Thus, in moderate oxidative stress induced by treatment with 0.5 mM CuSO_4_, toxic effects were observed for 5, 10 and 20 µg/mL EEP (Figure 2B). Concentrations of 50 and 100 µg/mL also enhanced copper-induced cell death. The highest applied concentration (100 µg/mL EEP) exhibited similar effects in physiological medium and in the presence of excess copper. The neurotoxic effect of small concentrations of EEP was particularly pronounced in the presence of 0.75 mM CuSO_4_. For example, 5 µg/mL EEP decreased survival by 93.2% in P19 neuronal cells which were concomitantly treated with 0.75 mM CuSO_4_ (from 28.8% to 2.0%), whereas in the presence of 0.5 mM CuSO_4_, cell survival was reduced by 32.2% (from 69.2% to 46.9%). As presented in Figure 2C, all investigated concentrations promoted copper-induced neuronal cell death in severe copper-induced oxidative stress, including concentrations in a range of 1–20 µg/mL that were not toxic *per se*. When oxidative stress was induced by exposure to 1 mM H_2_O_2_, 1–20 µg/mL of EEP were without any significant effects, whereas higher concentrations (50, 70 and 100 µg/mL) exacerbated H_2_O_2_-induced neuronal death (Figure 2D). 

### 2.2. Propolis Promoted ROS Generation and Caspase-3/7-Activity in P19 Neurons Exposed to Excess Copper

**Figure 3:**
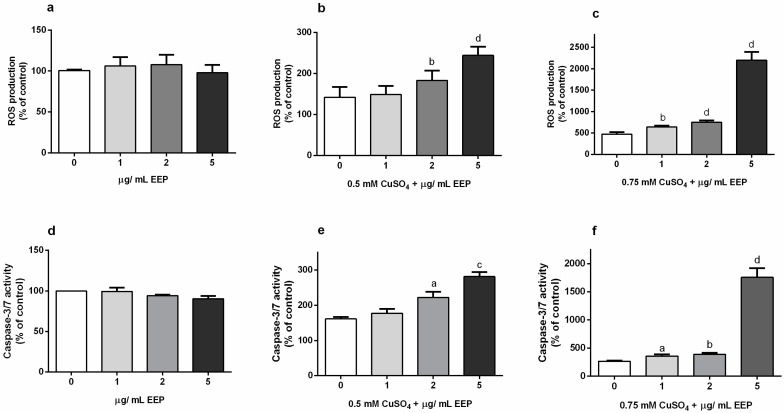
Effect of EEP on reactive oxygen species (ROS) production and caspase-3/7 activity in copper-induced oxidative stress in P19 neuronal cells. At 24 h from the beginning of exposure to EEP (1–5 µg/mL) and/or CuSO_4_ (0.5 and 0.75 mM), accumulation of ROS was quantified by an assay based on the generation of fluorescent products from the 2′,7′- dichlorofluorescin diacetate. In physiological conditions small concentrations of EEP did not affect intracellular ROS amounts (**a**), whereas in the presence of 0.5 and 0.75 mM CuSO_4_ (**b**,**c**, respectively), EEP stimulated production of dangerous oxidative species. EEP applied alone also did not modify basal level of caspase-3/7 activity (**d**), but induced caspase activation in the presence of copper ions (**e**,**f**). Data are expressed as mean ± SEM from 4–6 independent experiments performed in triplicate. Data were analyzed by one-way ANOVA followed by Dunnett’s *post* hoc test after exposure to 0.5 mM CuSO_4_ and by t-test after exposure to 0.75 mM CuSO_4_ (^a^ *P* < 0.05, ^b^ *P* < 0.01, ^c^ *P* < 0.001 and ^d^ *P* < 0.0001 versus copper-treated groups).

We used the cell permeable substrate, 2′,7′- dichlorofluorescin diacetate to monitor the effects of low concentrations of EEP (1–5 µg/mL) on ROS generation in physiological conditions (Figure 3A), and in a moderate and severe copper-induced oxidative environment. Data analysis revealed that 2 and 5 µg/mL of EEP significantly exacerbated ROS accumulation in the presence of 0.5 mM CuSO_4_ (Figure 3B). ROS levels were increased by 29% (2 µg/mL) and 72% (5 µg/mL) in comparison to cells exposed to 0.5 mM CuSO_4_. In the more severe oxidative environment, the increase in ROS generation was significant only for 5 µg/mL of EEP (one-way ANOVA and Dunnett’s test). In the presence of 0.75 mM CuSO_4_, 1 µg/mL EEP up-regulated ROS amounts by 35%, 2 µg/mL EEP by 58% and 5 µg/mL by 365%. According to statistical analysis, effects of all applied concentrations of EEP on ROS levels were statistically significant (*P* = 0.0012 for 1 µg/mL EEP; *P* < 0.0001 for 2 and 5 µg/mL EEP; unpaired t-test). 

As up-regulation of ROS mediates caspase activation, and caspase activation is implicated in certain models of neuronal cell death [20,21], we tested whether the pro-oxidant activity of EEP could result in an increased activation of executioner caspases-3/7. The results show that in physiological conditions, EEP does not modulate caspase-3/7 activity (Figure 3D), but it did promote caspase activation in a dose-dependent manner in the presence of 0.5 mM CuSO_4_ (Figure 3E). Statistical analysis by one-way ANOVA followed by Dunnett’s test indicated a significant effect of only 5 µg/mL in severe oxidative injury (Figure 3F), whereas data analysis using t-test indicated a significant effect of all applied concentrations on caspase-3/7 activity (*P* = 0.011 for 1 µg/mL, *P* = 0.002 for 2 µg/mL and *P* < 0.001 for 5 µg/mL). Taken together, obtained results suggest that the apoptosis-inducing effect of EEP due to increased ROS production probably underlies detrimental effects of EEP on neuronal viability. 

### 2.3. EEP Promoted Copper-Induced Up-Regulation of Bax and p53 Gene Expressions, Stimulated Transcription of GAPDH mRNA and Reduced Copper-Promoted Expression of c-fos Gene

**Figure 4:**
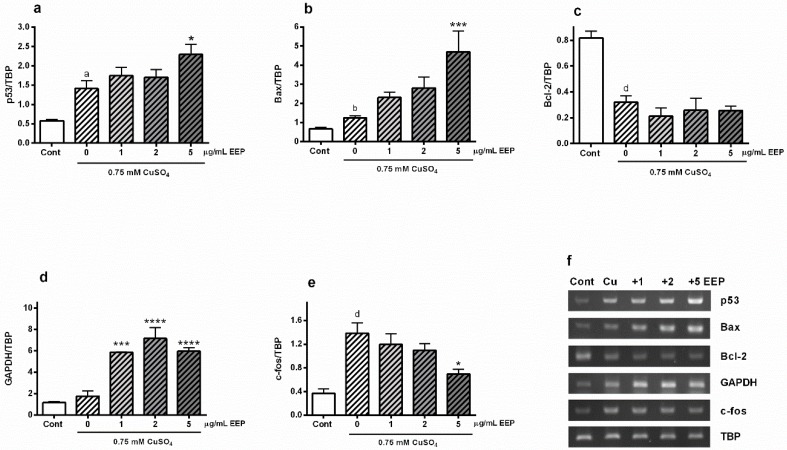
Effects of EEP on the expression of p53, Bax, Bcl-2, GAPDH and c-fos mRNA in copper-induced oxidative stress. At 24 h from the beginning of concomitant treatment with 0.75 mM CuSO_4_ and EEP (1–5 µg/mL), transcriptional expression of p53, Bax, Bcl-2, GAPDH and c-fos was determined by semiquantitative RT-PCR method. Total RNA from treated P19 neurons was extracted and reverse transcribed into cDNA. cDNA was further amplified by using specific primers and band intensities were normalized to the expression of the housekeeping gene TATA-box binding protein (TBP). Exposure to copper up-regulated expression of p53 (**a**) and Bax (**b**) mRNAs, and EEP further promoted transcription of these pro-apoptotic genes. EEP did not affect copper-induced decrease of Bcl-2 gene expression (**c**). In the presence of EEP, expression of GAPDH gene was highly promoted (**d**). Copper also induced expression of c-fos gene, and this effect was significantly reduced in the presence of EEP (**e**). Data are expressed as mean ± SEM from at least 6 independent RT-PCR analysis following three independent total RNA isolation. Data were analyzed by one-way ANOVA followed by post hoc Tukey’s test (^a^ *P* < 0.05, ^b^ *P* < 0.01 and ^d^ *P* < 0.0001 versus control; * *P* < 0.05, *** *P* < 0.001 and **** *P* < 0.0001 versus copper-treated group). Representative agarose gels are shown in (**f**).

The transcription factor p53 has an important role in the regulation of oxidative-stress driven neurodegeneration [22]. Hence, we analyzed the effect of EEP on copper-mediated stimulation of p53 mRNA expression. As we previously reported, exposure to excess copper promotes p53 gene expression [5]. In P19 neurons exposed to 0.75 mM CuSO_4_, p53 mRNA was up-regulated by 143% compared to control group, whereas in the presence of 5 μg/mL EEP, an additional increase of 63% was observed (Figure 4A). We also examined changes in Bax expression as Bax is a transcriptional target of p53 with a crucial role in membrane permeabilization and pro-apoptotic release of death mediators. Expression of Bax mRNA was almost doubled (86%) following copper treatment, and 5 μg/mL EEP markedly promoted up-regulation of Bax mRNA expression by 276% (Figure 4B). On the other hand, copper-induced decrease in the expression of anti-apoptotic protein Bcl-2 was not affected by EEP co-treatment (Figure 4C). We also looked for changes in the expression of glyceraldehyde-3-phosphate (GAPDH), a metabolic enzyme that may sense oxidative stress and participate in cell death events after nuclear translocation [23]. Exposure to 0.75 mM CuSO_4_ did not affect transcriptional level of GAPDH, whereas EEP strongly up-regulated expression of GAPDH. Statistically, the maximal effect was achieved at 1 µg/mL EEP (Figure 4D). Finally, we analyzed the expression of c-fos mRNA, an immediate early oxidative stress response gene [24]. As represented in Figure 4E, copper highly promoted c-fos expression (more than 2.5-fold), whereas the presence of EEP prevented c-fos up-regulation.

**Figure 5:**
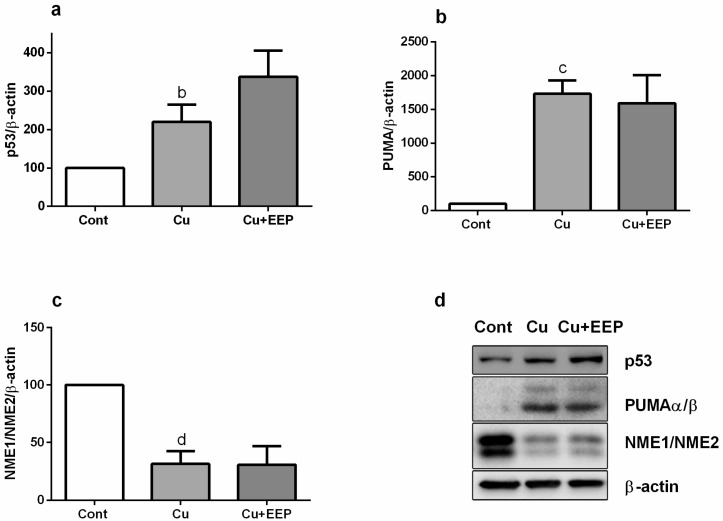
Effects of EEP on the expression of p53, PUMAα/β and NME1/NME2 in copper-induced oxidative stress. 24 h from the beginning of treatment with 5 µg/mL EEP and 0.75 mM copper, proteins (40 µg for p53; 20 µg for PUMAα/β and NME1/NME2) of total cell extracts were separated on 11% SDS polyacrylamide gel electrophoresis and electrophoretically transferred to nitrocellulose a membrane. Blots were probed with primary antibodies, followed by the appropriate horseradish peroxidase-labelled secondary antibody. Immunoreactivity was detected using enhanced chemiluminescence. β-actin was used as loading control for normalization. Data are expressed as mean ± SEM from up to four immunoblots of three independently prepare cell lysates. Western analysis revealed copper-induced increase in (**a**) p53 (^b^ *P* < 0.01), (**b**) PUMA α/β (^c^ *P* < 0.001) and (**c**) NME1/NME2 (^d^ *P* < 0.01) protein expressions. Following densitometric analysis, obtained data were analyzed with one-way ANOVA followed by Tukey’s test. Representative Western blots are presented in (**d**).

Puma, a downstream p53 target, has been identified as an important regulator of Bax activation and neuronal apoptosis during oxidative stress [22]. Thus, we analyzed the expressions of p53 and Puma at the protein level. We detected approximately a threefold increase of p53 amount in copper-induced oxidative stress (Figure 5A) and a striking up-regulation of Puma expression (Figure 5B). However, neither of these effects were affected by EEP treatment, although there was a trend toward increase in the expression for p53. Similarly, copper induced marked decrease in the expression of NME1/NME2 proteins, which may limit oxidative stress in mammalian cells [25], but the presence of EEP did not modify the copper-mediated down-regulation of NME1/NME2 expression at the protein level (Figure 5C). 

### 2.4. Neurotoxic Effects of EEP in The Presence of Excess Copper was Prevented by SB203580, The p38 Kinase Inhibitor, and Exacerbated by SP6000125, the JNK Pathway Inhibitor 

**Figure 6:**
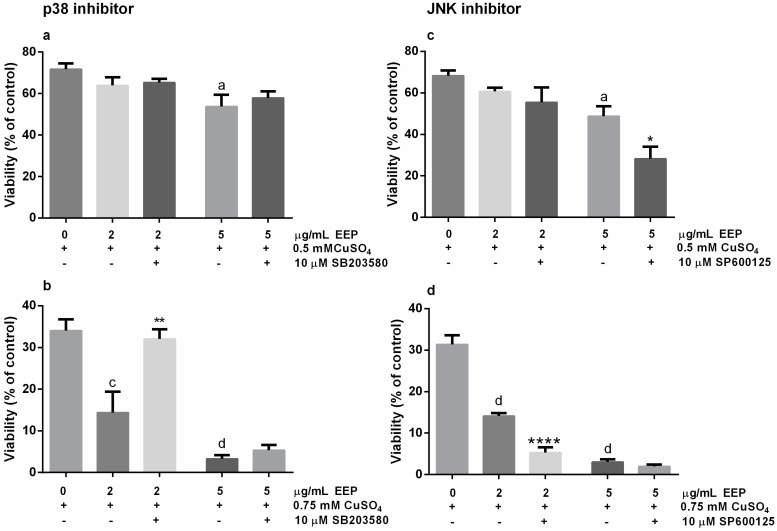
Modulation of copper-induced neurotoxic effects of EEP by the p38 inhibitor SB203580 and JNK inhibitor SP600125. P19 neuronal cells were treated with 10 µM SB203580 or 10 µM SP600125 for 1 h prior to and during the concomitant treatment with copper (0.5 and 0.75 mM) and EEP (2 and 5 µg/mL). At 24 h from the beginning of treatment with copper and EEP, neuronal cell viability was determined by the MTT assay. In moderate oxidative injury, SB203580 did not modify neuronal cell viability (**a**), whereas in severe oxidative conditions, toxic effects of 2 μg/mL EEP was prevented by the p38 inhibitor (**b**). Detrimental effects of the inhibition of JNK signaling were evidenced in neuronal cells exposed to both 0.5 mM (**c**) and 0.75 mM CuSO_4_ (**d**). Values are expressed as means ± SEM from 5 independent experiments performed in quadruplets. Data were analyzed by one-way ANOVA followed by post-hoc Tukey’s test (^a^ *P* < 0.05, ^c^ *P* < 0.001 and ^d^ *P* < 0.0001 versus copper treated group; * *P* < 0.05, ** *P* < 0.01 and **** *P* < 0.0001 versus copper + EEP-treated group).

The p38/p53 signaling may contribute to apoptotic response in oxidative stress-induced neuronal cell death [26]. To determine whether p38 MAP kinase was involved in p53-mediated neuronal cell death, we treated P19 neuronal cells with CuSO_4_ along with 2 or 5 μg/mL EEP and in the presence/absence of SB203580, a p38 inhibitor. In mild oxidative stress induced by exposure to 0.5 mM CuSO_4_, effect of 10 µM SB203580 on EEP-induced reduction of neuronal viability was not detected by the MTT assay (Figure 6A). On the contrary, in severe copper-induced oxidative conditions, SB203580 was able to completely prevent the toxic effects of 2 µg/mL of EEP (Figure 6B). 

The c-Jun N-terminal kinases (JNK) is also activated in response to oxidative injury and may affect survival outcome [27]. In P19 neuronal cells exposed to 0.5 mM CuSO_4_, a significant pro-death effect of SP600125, a JNK inhibitor, was observed for 5 μg/mL EEP (Figure 6C). In severe oxidative injury induced by treatment with 0.75 mM CuSO_4_, SP600125 promoted additional decrease in the viability of neuronal cells treated with 2 μg/mL EEP (Figure 6D). Altogether, these results suggest that depending on the severity of neuronal damage and the applied concentration of EEP, effects of EEP on the reduction of neuronal survival are, at least in part, mediated through p38 and JNK signaling in P19 neuronal cells.

### 2.5. Quercetin Induced Similar Effects as Propolis and Promoted Copper-Induced Death of P19 Neuronal Cells

**Figure 7:**
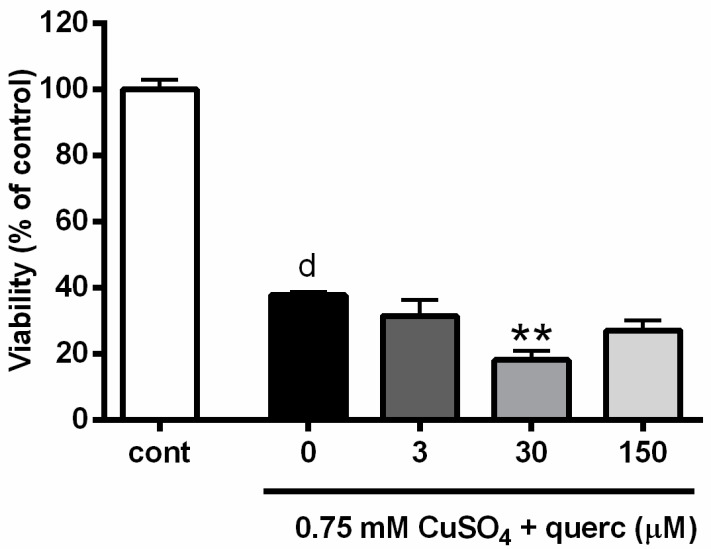
Quercetin exacerbated copper-induced neuronal death in P19 cells. P19 neuronal cells were exposed to different concentrations of quercetin (3, 30 and 150 µM) and 0.75 mM CuSO_4_. Neuronal viability was determined 24 h from the beginning of treatment using the MTT assay. Quercetin-augmented copper-induced neuronal death at 30 µM. Data are expressed as means ± SEM from five independent experiments performed in quadruplets. ^d^ *P* < 0.0001 versus control group; ^**^ *P* < 0.05 versus Cu + EEP group (one-way ANOVA followed by post hoc Tukey’s test).

Quercetin glycosides represent the important flavonoid fraction found in propolis [28]. Hence, we analyzed the effects of quercetin during severe copper-induced oxidative stress in P19 neuronal cells to see if we could observe effects that are similar to those of EEP. We exposed P19 neuronal cells to 0.75 mM CuSO_4_ concomitantly with 3, 30 or 150 µM (1, 10 and 50 µg/mL) quercetin. We applied this range of quercetin concentrations based on previous studies, where we evaluated the neuroprotective potential of quercetin against mild and severe hydrogen-peroxide induced oxidative stress in P19 neuronal cells [29,30]. As determined by MTT assay and represented in Figure 7, 30 µM quercetin augmented neurotoxic effects of copper and reduced survival of P19 neuronal cells from 35.4 to 18.3%. Similar results were obtained using trypan blue staining (data not shown).

## 3. Discussion

Our study revealed that EEP at small, *per se*, non-toxic concentrations, exacerbate copper-induced death of P19 neuronal cells. EEP augmented ROS production and promoted caspase-3/7 activity. The detrimental effect of EEP was prevented in the presence of the p38 inhibitor; suggesting that pro-oxidative and p38-mediated pro-apoptotic mechanisms contributed to the toxic outcome of EEP treatment. Although neuroprotective actions of propolis have been reported [17,31], its antiproliferative and cytotoxic effects are well-documented in different human cancer cells [32,33,34,35].

The chemical composition of propolis is very complex and varies with the season and the flora of the region from which it originates. It contains at least 300 compounds of which more than 100 can be found in any given sample [14]. The bioactive components of propolis include fatty, aliphatic and aromatic acids, esters, flavonoids, aromatic aldehydes, beta-steroids and terpenes, among others [17,18,32]. Previous analysis of the Croatian propolis sample revealed that EEP contained 40.2% of total flavonoids, including quercetin [36]. In this study, the authenticity of the propolis was confirmed by measuring FTIR spectra (Figure 1). In general, there is a nice agreement in peak positions with the spectrum reported in Franca et al. [19]. Differences in relative intensities are most likely due to the different origin of the propolis samples. However, if compared with the spectra of flavanols as presented and interpreted by Baranovic and Segota [37], one is prompted to conclude that the main characteristics of the 1700–1000 cm^−1^ region can be attributed to the flavanols that have been detected in propolis by other methods [38]. 

It has been recognized that propolis may induce DNA damage in the presence of transition ions. Flavonoids that are commonly found in different propolis samples are also recognized as DNA-damaging agents. Accordingly, it is proposed that flavonoids in propolis act as electron carriers from transition metals to molecular oxygen, thus, generating superoxide anion and H_2_O_2_, which are responsible for direct pro-oxidant actions and harmful effects on viability [39].

Increased generation of ROS leads to depletion of endogenous antioxidant defense and induces oxidative stress. The transcription factor p53 has an important role in neuronal response to oxidative injury. Rapid increase in p53 levels and stimulation of its activity are involved in neuronal cell death in various types of oxidative damage, including exposure to excess copper [5,40,41,42]. Bax, a pro-apoptotic member of the Bcl-2 family, is a transcriptional target of p53, and in p53-mediated neuronal cell death, Bax is required for caspase-3 activation [20]. PUMA is another transcriptional target of p53 with important role in neuronal cell death during oxidative stress. Puma associates with Bax and promotes its activation and may inactivate all anti-apoptotic members of the Bcl-2 family [43]. 

In P19 neuronal cells exposed to copper, we found an increased expression of p53, Bax and PUMA, thus, confirming the p53-dependent, pro-apoptotic mechanism of copper-provoked neuronal cell death (Figure 4). Expression of p53 and Bax mRNA was further augmented in the presence of EEP. In the presence of a DNA damaging stimulus, such as oxidative stress, transcription factor NF-κB may induce transcription of p53 gene, whereas in turn p53 regulates expression of Bax gene. NF-κB can regulate expression of p53 directly via κB sites in the promotor sequence of the p53 gene [44]. Although p53 increased at the protein level, it did not reach any statistical significance (Figure 5A). Nonetheless, the observed trend of up-regulation, taken together with the p53 mRNA enhancement, strongly suggests that p53-mediated pro-apoptotic mechanisms could be involved in neurotoxic effects of EEP. In general, the diversity of p53-mediated responses is determined by selective transactivation of target genes depending on the severity of stress stimulus. Besides promoting expression of pro-apoptotic genes, p53 may induce apoptotic effects through transcriptional inhibition of survival genes such as Bcl-2 [40]. We indeed observed that copper-induced cell death was associated with decreased expression of Bcl-2, but similarly to PUMA, EEP did not further affect levels of Bcl-2 mRNA. Previous studies have revealed that in a concentration-dependent manner, propolis and its major compounds may reduce survival by inducing either a p53-dependent cell death, which is accompanied by up-regulation of p53 and its downstream targets, or a p53-independent death without changes in the gene expression profile of p53 [33,34,35,45]. Such findings indicate a complicated and heterogeneous nature of cellular and molecular events that could be triggered by propolis and result in decreased cellular viability. 

Yet, another transcriptional target of p53 whose upregulation and nuclear translocation has been found in neurons following apoptotic stimuli is GAPDH [40,46]. A growing body of evidence suggests an important role of GAPDH in sensing oxidative stress and induction of neuronal cell death. GAPDH may stimulate p53 expression and phosphorylation, and activate p53-mediated cell death cascade [23]. In P19 neuronal cells, the toxic effects of EEP were accompanied by the up-regulation of both p53 and GAPDH mRNA, which is fascinating and certainly warrant further studies to reveal if EEP-induced GAPDH up-regulation contributes to neuronal death through p53/GAPDH-mediated mechanism. 

Detrimental effects of EEP on the survival of P19 neuronal cells were associated with prominent ROS increase and resulted in the up-regulation of caspase-3/7 activity (Figure 3). Part of the observed increase in ROS levels could be attributed to direct ROS production through the Fenton chemistry. On the other hand, crosstalk between p53 and ROS, and interdependence of their signalling pathways may also have a strong impact on redox homeostasis and ultimately neuronal cell viability [47]. Namely, p53 has an important role in the regulation of cellular ROS production, whereas ROS in turn may modulate transactivation of p53 target genes. Besides typical pro-apoptotic proteins such as Bax and PUMA, among p53-induced genes are ROS-generating enzymes whose up-regulation may increase ROS levels, and hence, contribute to oxidative stress, caspase-3/activation and apoptosis. In addition, an alternative way to enhance ROS production could be through p53-mediated suppression of antioxidant genes [48]. Taken together, we suggest that EEP-induced increase of ROS production could be elicited, at least in part, via p53-dependent mechanisms; a mechanism that was also observed in cancer cells [49]. 

In postmitotic neurons, MAPK have an important role in intracellular signalling during oxidative stress-response and apoptosis [50]. Depending on the cell type and type of the injury, activation of MAPK cascades can promote either neuronal cell death or survival. MAPKs mediate their effects through phosphorylation of specific effector proteins, mostly transcription factors [27]. For example, in polychlorinated biphenyls (PCBs)-induced toxicity, downregulation of ERK2 phosphorylation resulted in enhanced expression of repressor element-1 silencing transcription factor (REST), leading to neuronal death [51]. In neurons exposed to methylmercury (MeHg), increased phosphorylation of p38 was involved in up-regulation of REST, histone deacetylase 4 (HDAC4) and specificity protein 1 (Sp1) and Sp4 expression that ended in brain-derived neurotrophic factor (BDNF) reduction and neuronal death. Further studies revealed that microRNA (miR)206 regulated REST and Sp4 at transcriptional level through the activation of the transcription factor JunD, a nuclear substrate of both JNK and ERK [52,53]. In neuronal cell death, MAPK may regulate apoptotic cascade through the specific phosphorylation of p53. Phosphorylation of p53 may increase its stability, which in turn may contribute to increase in p53 expression and transcriptional activity. Additionally, the p38 signalling pathway is often activated in response to toxic drugs and oxidative stress, and have an important role in p53 phosphorylation, enhancing its pro-apoptotic effects [26,40,54]. In P19 neuronal cells, we demonstrated a neuroprotective effect of SB203580 a p38 inhibitor. Our results indicate that an interplay between p53, ROS and p38 may contribute to EEP-provoked neuronal cell death in the presence of excess copper. Similarly, the importance of p53/p38 functional interactions was observed in UV-induced cellular stress [55]. More importantly, caffeic acid phenethyl ester (CAPE), one of the most important bioactive compounds of propolis, as well as propolis extracts, may activate the p38 pathway, induce p53 and Bax and initiate apoptosis in cancer cells [32]. In a vicious circle, p53-induced ROS may have an important role in p38 activation, whereas, p38-mediated phosphorylation of p53 preserves full activation of p53 and thus, ROS generation [54]. Accordingly, inhibition of p38 pathway was effective against apoptosis induced by a wide variety of oxidative stressors [50,56]. 

JNK is another MAPK kinase whose activation is increased in response to ROS [24]. In contrast to SB203580, effect of EEP on the copper-induced neuronal cell death was exacerbated in the presence of the JNK inhibitor SP600125. Thus, our results suggest that JNK activation is beneficial in neurons exposed to excess copper and EEP. Although JNK is usually perceived as an apoptosis-inducing protein, stress-related induction of JNK activity is still controversial. Different outcomes may be attributed to different JNK isoforms, which may have specific roles in stress responses and regulation of gene expressions. The protective role of JNK activations has been demonstrated in neuronal cells in several different death paradigms [57,58,59]. Oswald and co-authors [24] argued that JNK/AP-1 system is the most important adaptive response to ROS in neurons as it promotes transcription of antioxidant proteins. This is in agreement with our results, which demonstrates a protective effect of JNK activation in P19 neuronal cells. As AP-1 is a heterodimer composed of transcription factors Fos and Jun, perhaps the EEP-induced decrease in c-fos might contribute to decreased AP-1 activity, decreased ability to cope with oxidative stress, and ultimately, neurotoxic effects. In contrast to our results, protective effects of JNK inhibition were observed in oxidative stress-conditions in non-neuronal cells. The mechanism of this effect was at least in part mediated through the nucleoside diphosphate kinase activity of the NME1 protein (NDPK-A, Nm23-H1), which prevented JNK activation and reduced oxidative stress level [25]. In P19 neuronal cells, we observed a prominent reduction of NME1/NME2 protein expression levels in a copper-enriched environment. This might indicate an involvement of NME1/NME2 in JNK signaling and ROS generation in P19 neuronal cells exposed to copper and EEP, an effect that may require clarification in further studies. 

As mentioned previously, the flavanol quercetin and its derivatives are usually found in propolis samples of different geographical and seasonal origin [36,60,61]. In P19 neuronal cells, we observed that non-toxic concentration of quercetin may exacerbate toxic effects of copper in severe oxidative stress. Similar to propolis, quercetin is anticipated as a safe supplement that may improve human well-being by re-gaining redox homeostasis. Thus, our results are particularly interesting as they indicate a general neurotoxic potential of phytopharmaceutical in a copper-enriched environment. Considering that elevated levels of copper are found in the blood of patients with neurodegenerative diseases, this study also emphasizes that caution is required if prolonged supplementation with natural products is included as adjuvant therapy in these patients. Safety and efficacy are the two major concerns regarding the long-term usage of herbal formulations. Our study indicates that supplements appreciated by their antioxidant effects may switch to a prooxidant action in the presence of metal ions. *Ortho*-dihydroxyl groups, which are present in phenolic components of propolis, are able to chelate with Cu^2+^ and induce this pro-oxidant activity [62]. Detrimental effects of phenolic compounds such as quercetin, can be explained in paradoxical terms: they become toxic as a result of their protection. This means that, although ROS are neutralized, reactive oxidation products with toxic potentials are concomitantly formed [63]. Thus, in the presence of quercetin, Cu^2+^ can be reduced to Cu^+^, forming a semiquinone radical (semioxidized quercetin). This radical undergoes a second electron transfer reaction with molecular oxygen, generating ortho-quinone and superoxide anion (O_2_**^•^**). In the next step, superoxide anion reacts with catalytic Cu^+^ to give hydrogen peroxide, which is via a Fenton-type reaction converted into hydroxyl radical (**^•^**OH). Hence, re-oxidation of Cu^+^ to Cu^2+^ leads to the new generation of ROS [62]. If this mechanism underlies ROS generation in P19 neuronal cells, it is intriguing how this prooxidant cascade exerts stronger effects at small concentrations of EEP. As suggested by Filipe et al. [64], one possibility is that the prooxidant mechanism at higher concentrations is still operative, but less visible because the antioxidant activity (ROS scavenging, metal chelation) prevails.

## 4. Conclusions

For the first time, we report here the neurotoxic effects of ethanolic extract of propolis in the presence of excess copper ions. We suggest that the underlining mode of action of its detrimental effects is related to p53-mediated mechanisms and Fenton chemistry that promote ROS generation and p38 activation, thus leading to caspase-3/7 activation and initiation of an apoptotic cascade. Further studies are needed to confirm these neurotoxic effects of EEP in conditions of copper overload in animals and human subjects and to reveal, in more detail, the molecular mechanisms of its toxicity and possible pathological outcomes of prolonged propolis consumption.

## 5. Materials and Methods 

### 5.1. Chemicals

*All-trans* retinoic acid (ATRA), cytosine-arabinofuranoside (AraC), 2′7′-dichloro-fluorescein diacetate (DCF-DA), 3-(4,5-dimethylthiazol-2yl)2,5-dyphenyl-2H-tetrazolium bromide (MTT), hydrogen peroxide solution and quercetin dihydrate were purchased from Sigma-Aldrich Chemicals (St. Louis, MO, USA). Culture medium (Dulbecco’ Modified Eagle’s Medium (DMEM)), foetal bovine serum (FBS), trypsin, antibiotics, L-glutamine and supplements for differentiation of P19 cells were also purchased from Sigma-Aldrich (St. Louis, MO, USA) or Gibco (Paisley, UK). SP600125 and SB203580 were from Alfa Aesar (Ward Hill, MA, USA). EEP was obtained as previously described [57]. All other chemical used were of analytical grade.

In order to confirm the authenticity of the propolis, infrared spectra of EEP were recorded with an ABB Bomem Mb102 Fourier transform infrared (FTIR) spectrometer at room temperature. For each infrared spectrum, 30 interferograms were collected with a nominal resolution of 4 cm^−1^. The reference spectra were recorded under the same conditions. The spectra were collected on a MKII Golden Gate single reflection attenuated total reflection (ATR) accessory.

### 5.2. P19 Cell Culturing and P19 Neuronal Differentiation

Undifferentiated P19 cells were grown in DMEM medium (high-glucose) supplemented with 10% heat-inactivated FBS, 2 mM L-glutamine, 100 units/mL penicillin G and 100 µg/mL streptomycin (growth medium) in a humidified atmosphere of 5% CO_2_ at 37 °C.

#### 5.2.1. Neuronal Differentiation of P19 Cells

##### P19 Embryonal Body Formation (DIV 0-4)

Neuronal differentiation of P19 cells was induced by exposure to 1 µM ATRA for 4 days. Individual cells (1 × 10^6^) were seeded in 10 cm bacteriological grade Petri dishes containing 10 mL of induction medium (DMEM supplemented with 5% FBS, 2 mM L-glutamine and antibiotics). ATRA was added immediately after plating. Aggregates (embryonal bodies) of P19 cells were formed after 1–2 days. After two days, the media was changed by transferring suspension to a 15 mL conical tube. 

Aggregates were allowed to settle and after removal of old media, they were resuspended in fresh medium containing ATRA and returned to the same Petri dish. Aggregates were grown in a humidified atmosphere for two more days.

##### P19 Neuronal Differentiation (DIV 4-8)

After the fourth day of ATRA treatment, P19 cells were plated on treated tissue-culture plastics for complete neuronal differentiation and maturation. P19 embryonal bodies were harvested, washed twice with phosphate buffer saline (PBS) and resuspended in 2 mL of 0.05% trypsin-1mM EDTA in PBS. Cells were incubated for 10 min, dissociated by pipetting after addition of 5 mL of growth medium, collected by centrifugation (1250 rpm, 5 min) and resuspended in growth medium. For optimal neuronal differentiation, single cells at a density of 10^5^ cells/cm^2^ were plated and grown in growth medium for 2 days. Since serum inhibits neuronal production and favors the growth of astrocytes and fibroblasts, after 2 days the medium was changed to serum-free differentiation medium containing DMEM supplemented with insulin, transferrin, selenium and ethanolamine solution (ITS-X, Gibco), 0.5 mM L-glutamine and antibiotics (neuron-specific medium) and cells were differentiated for additional 2 days. As P19 cells differentiate into a mixed population of neurons, fibroblasts and astrocytes, mitotic inhibitor AraC was added at 10 μM concentration to inhibit the proliferation of non-neuronal cells. Complete neuronal maturation of P19 neurons was confirmed by immunofluorescence staining against neuron specific marker β-III tubulin, 8 days after the initiation of ATRA treatment [21].

### 5.3. Drug Treatment

To examine the effects of propolis on copper-induced neuronal cell death, we used P19 neuronal cells at DIV8. Each batch of cultured cells was divided into control and drug treated groups. For dose-response studies, cells were exposed to different concentrations of EEP for 24 h. Stock solution of EEP was prepared in DMSO (100 mg/mL) and diluted with DMEM medium to final concentrations (1–100 μg/mL). Activation of JNK and p38 signaling was investigated using SP6000125 and SB203580, respectively. SP6000125 is an inhibitor of c-Jun N-terminal kinase (JNK), whereas SB203580 was used to elucidate the role of p38 signaling. Both inhibitors were present for 1 h prior and during the exposure to EEP in combination with 0.5 and 0.75 mM CuSO_4_.

### 5.4. Assessment of Cell Death 

To evaluate cell survival after exposure to EEP and CuSO_4_, we used a colorimetric MTT assay. The estimation of neuronal cell viability was based on the ability of NAD(P)H-dependent oxidoreductase enzymes to reduce MTT to water-insoluble purple formazan crystals, proportional to the number of metabolically active cells. P19 neurons (DIV4) were plated in 96-well culture plates at a density 30 × 10^3^ cells/well, fully differentiated for additional 4 days and then treated. At the end of the treatment period, 0.5 mg/mL MTT in neuron-specific medium (P19 cells) was added (40 μL/well) and incubated for 3 h at 37 °C. The formazan dye was dissolved by adding 160 μL of dimethyl sulfoxide (DMSO) and the absorbance was recorded with an automatic microplate reader at 570 nm.

### 5.5. Detection of ROS Levels

Intracellular accumulation of ROS as an indicator of general oxidative stress level was determined by the routinely used assay that utilizes lipophilic probe 2′,7′- dichlorofluorescin diacetate, DCF-DA. This non-fluorescent compound accumulates within cells, and after deacetylation by intracellular esterases reacts with endogenous ROS forming highly fluorescent product dichlorofluorescein (DCF). To evaluate ROS generation, P19 neurons (DIV4) were plated in 96-well white plates (Nunc) at a density of 30 × 10^3^ cells/well in 100 μL of complete growth medium, differentiated and treated at DIV8 with CuSO_4_ and EEP. At 24 h after treatment, P19 neurons were incubated with 100 μM DCF-DA in PBS for 1 h, rinsed with PBS, and incubated for an additional 1 h in PBS. Oxidation of DCF-DA to DCF was measured on a plate reader (Fluoroskan Ascent FL, Thermo Scientific, Waltham, MA, USA) at the excitation wavelength of 485 nm, and emission wavelength of 538 nm. The detected fluorescent intensities were normalized to the number of viable cells.

### 5.6. Determination of Caspase -3/7 Activity

Activity of caspase-3/7 enzymes was measured using the Apo-ONE^®^ Homogeneous Caspase-3/7 assay (Promega, Madison, WI, USA). These two members of the caspase family are the key effector enzymes in caspase-dependent apoptosis. To perform the assay, an optimized cell permeabilization buffer and non-fluorescent caspase substrate Z-DEVD-rhodamine 110 were mixed (50 µL) and added directly to treated P19 neurons grown in equal amount of the culture medium. The sample was shaken for 5 min and then incubated at RT in the dark for 4 h. Cleavage of the DEVD peptides by activated caspase-3/7 releases the rhodamine 110 which becomes intensely fluorescent when excited. The amount of generated fluorescent product is proportional to the amount of caspase-3/7 cleavage activity in each sample. The plate was read on a plate reader (Fluoroskan Ascent FL, Thermo Scientific, Waltham, MA, USA) at the excitation wavelength of 485 nm, and emission wavelength of 538 nm.

### 5.7. Determination of mRNA Levels by Semi-Quantitative RT-PCR Method

Expressions of Bcl-2, Bax, c-fos, p53, and GAPDH at the transcriptional level were examined by semiquantitative RT-PCR analysis according to the method previously described [29]. Briefly, cDNAs were amplified and analyzed during the two consecutive cycles in the log phase of PCR reactions. PCR primers, annealing temperatures, and numbers of cycles are listed in Table 1. The reactions were performed in a Perkin Elmer 9600 thermocycler and amplified products (10 µL) were electrophoretically separated on a 1.5% agarose gel and stained with ethidium bromide (0.5 µg/mL). Optical densities of detected bands were analyzed with ImageJ NIH software 1.0. Expression of the housekeeping gene TATA-box binding protein (TBP) mRNA was used as an internal standard for normalization.

### 5.8. Western Blot Analysis of p53, PUMA and NME1/2 Expression

The expression of proteins was analyzed in total cell extracts. Following treatment, P19 neurons grown in 25 cm^2^ flasks were washed twice with PBS, lysed by scrapping into 100 μL of RIPA buffer (Sigma Aldrich, St. Louis, MO, USA) containing protease inhibitors (Complete, Mini, EDTA-free Protease Inhibitor Cocktail Tablets; Roche, Indianapolis, IN, USA) and sonicated. The protein concentration was determined using the Pierce BCA Protein Assay kit (Thermo Fisher Scientific, Waltham, MA, USA). A standard curve was developed using a series of bovine serum albumin dilutions in the 100 μg/mL to 1500 μg/mL range, and a linear plot of concentration vs. absorbance (at 570 nm) was generated to determine protein concentration in different samples. Equal amounts of proteins were boiled for 2 min, loaded on 11% sodium dodecyl sulphate-polyacrylamide gels, separated by electrophoresis and transferred to nitrocellulose membranes (Whatman, GE Healthcare, Life Sciences, Berlin, Germany). Non-specific binding was blocked by 5% non-fat milk in Tris-buffered saline containing 0.05% Tween 20 (TBST) for 1 h at RT. Membranes were incubated overnight with the primary antibody and then for 2 h at RT by appropriate secondary antibody. 

A total of 40 µg of proteins were loaded for p53 detection, and 20 µg for PUMA and Nme1/Nme2 detection. Blots were incubated with primary anti-p53 antibody (clone Pab 1801: sc-98; Santa Cruz Biotechnology; 1:500), anti-PUMAα/β antibody (G-3, sc-374223; Santa Cruz Biotechnology; 1:500) and anti NME1/NME2 antibody (kindly provided by I. Lascu and S. Volarević; 1:3000). Anti-β-actin antibody (AC-15, Sigma-Aldrich; 1:5000) was used for normalization. Peroxidase-labelled anti-mouse IgG (Amersham ECL mouse IgG; 1:5000) or anti-rabbit IgG (Cell Signaling Technology; 1:3000) were used as secondary antibodies. All immunoblots were processed using the Western Lightning^®^ Plus-ECL Enhanced Chemiluminescence Substrate (PerkinElmer, Waltham, MA, USA). Relative intensities of the bands were quantified by using ImageJ NIH software after detection on Alliance 4.7 (UVItec Cambridge, London, UK).

### 5.9. Statistical Analysis

Data are presented as the mean ± standard error mean (SEM). Statistical comparisons were made by t-test or one-way analysis of variance (ANOVA) followed by Dunnett’s or Tukey test using GraphPad Prism software. The normality of the data distribution was verified using the D’Agostino-Pearson omnibus test. A *P* < 0.05 was considered statistically significant.

## Figures and Tables

**Table 1:** Primer sequences and conditions used for PCR amplifications.

Gene	Primer Sequence (5′ → 3′)	Product Length (bp)	Annealing Temp. (°C)	Number of Cycles
Bcl-2	F: GGAGATCGTGATGAAGTACATAC R: CCTGAAGAGTTCCTCCACCACC	373	58	27–28
Bax	F: ATCGAGCAGGGAGGATGGCT R: CTTCCAGATGGTGAGCGAGG	470	62	27–28
c-fos	F: GAATGGTGAAGACCGTGTCAGG R: CGTTGCTGATGCTCTTGACTGG	456	60	24–25
p53	F: AGAGACCGCCGTACAGAAGA R: CTGTAGCATGGGCATCCTTT	231	62	31–32
GAPDH	F: ACCACAGTCCATGCCATCAC R: TCCACCACCCTGTTGCTGTA	452	60	19–20
TBP	F: ACCCTTCACCAATGACTCCTATG R: ATGATGACTGCAGCAAATCGC	190	60	26–27
